# Supplementary material for: MetaRibo-Seq measures translation in microbiomes
Source: Nat Commun. 2020 Jun 29;11:3268. doi: 10.1038/s41467-020-17081-z (PMC7324362; doi:10.1038/s41467-020-17081-z)
Supplement: Supplementary file 10 — Supplementary Data 7 [file 41467_2020_17081_MOESM10_ESM.zip › File2/Confidence_High_Taxonomy/114093_out.krona.html]

Javascript must be enabled to view this page.

members
magnitude
magnitudeUnassigned
count
unassigned
taxon
rank

114093\_out

2

2
2
superkingdom

1
phylum
1239

1
class
186801

186802
order
1

186806
1
family

1730
genus
1

39485

SRS017821\_contig\_number\_contig-100\_778.145592
1
species

1
phylum
544448

31969
class
1

186329
order
1

1
family
2146

genus
1
2147


SRS058070\_contig\_number\_contig-100\_3108.136618
species
1
1262686
